# Bioprospecting of Novel Extremozymes From Prokaryotes—The Advent of Culture-Independent Methods

**DOI:** 10.3389/fmicb.2021.630013

**Published:** 2021-02-10

**Authors:** Maksim Sysoev, Stefan W. Grötzinger, Dominik Renn, Jörg Eppinger, Magnus Rueping, Ram Karan

**Affiliations:** ^1^KAUST Catalysis Center (KCC), King Abdullah University of Science and Technology (KAUST), Thuwal, Saudi Arabia; ^2^Institute for Experimental Molecular Imaging, University Clinic, RWTH Aachen University, Aachen, Germany

**Keywords:** extremophile, culture-independent methods, metagenomics, SAG, halophiles, thermophiles, psychrophile

## Abstract

Extremophiles are remarkable organisms that thrive in the harshest environments on Earth, such as hydrothermal vents, hypersaline lakes and pools, alkaline soda lakes, deserts, cold oceans, and volcanic areas. These organisms have developed several strategies to overcome environmental stress and nutrient limitations. Thus, they are among the best model organisms to study adaptive mechanisms that lead to stress tolerance. Genetic and structural information derived from extremophiles and extremozymes can be used for bioengineering other nontolerant enzymes. Furthermore, extremophiles can be a valuable resource for novel biotechnological and biomedical products due to their biosynthetic properties. However, understanding life under extreme conditions is challenging due to the difficulties of *in vitro* cultivation and observation since > 99% of organisms cannot be cultivated. Consequently, only a minor percentage of the potential extremophiles on Earth have been discovered and characterized. Herein, we present a review of culture-independent methods, sequence-based metagenomics (SBM), and single amplified genomes (SAGs) for studying enzymes from extremophiles, with a focus on prokaryotic (archaea and bacteria) microorganisms. Additionally, we provide a comprehensive list of extremozymes discovered via metagenomics and SAGs.

## Introduction

A mounting paradigm shift toward using sustainable resources has stimulated exploring new efficient approaches in technological processes (Raddadi et al., 2015; Lamers et al., 2016; Krüger et al., 2018). Enzymes, as natural catalysts, have shown remarkable abilities that have revolutionized the chemical, biotechnological, bioremediation, agricultural, and pharmaceutical industries (Martin and Vandenbol, 2016; Wiltschi et al., 2020). However, the narrow range of stability of most described biocatalysts from mesophilic organisms limits their use for many applications (Raddadi et al., 2015). Enzymes derived from microorganisms thriving under harsh conditions, called extremophiles, can overcome these restrictions, and today, such biocatalysts are in higher demand than ever before (Karan et al., 2012a; Rizk et al., 2012; Johnson, 2014; Raddadi et al., 2015; Sarmiento et al., 2015; Singh et al., 2016; Jorquera et al., 2019). The diversity of extreme environments promises to reveal biocatalysts capable of withstanding harsh industrial conditions, providing better efficiency with the lower environmental burden (Elleuche et al., 2014; Grötzinger et al., 2017). Extremophiles are present in all three domains of life (bacteria, archaea, and eukarya) (Rothschild and Mancinelli, 2001; Jorquera et al., 2019). According to their natural habitats, extremophiles are classified into thermophiles, alkaliphiles, acidophiles, halophiles, and others (Karan et al., 2012a, b; Raddadi et al., 2015). Therefore, compared to their mesophilic equals, extremozymes are usually able to perform reactions under a broader range of conditions (Karan et al., 2011; Reed et al., 2013; Sarmiento et al., 2015). This extended activity range often allows extremophiles to identify as polyextremophiles, as they tolerate multiple extreme conditions (Karan and Khare, 2010; Rekadwad and Khobragade, 2017; Karan et al., 2019). Therefore, polyextremophiles are perfect candidates as a source of novel enzymes for industrial needs (Raddadi et al., 2015; Sarmiento et al., 2015; Lamers et al., 2016; Krüger et al., 2018).

However, only a small percentage of all microorganisms can be grown in a laboratory environment. This is particularly the case for archaea, representing a large group of prokaryotic extremophiles (Kaeberlein et al., 2002; Zengler et al., 2002; Buerger et al., 2012). Improving computing and bioinformatics technologies have made it possible to study the “dark matter” of the microbial world by looking at the genome data extracted from microbial habitats (Hedlund et al., 2014).

The advent of next-generation sequencing allowed researchers to sequence thousands of microorganisms in parallel, and due to the high sensitivity of these genome-based methods, now we can study microbes with a very low abundance that may be overlooked by other methods (Grötzinger et al., 2014; Laurence et al., 2014; Karan et al., 2020). Accordingly, sequence-based metagenomics (SBM) was born, which studies microorganisms by randomly shearing environmental DNA, sequencing it, and assembling the reads (Hugenholtz and Tyson, 2008).

Further advances allowed researchers to study the genome from single cells (Grötzinger et al., 2014, 2017; Akal et al., 2019; Karan et al., 2019; Vogler et al., 2020). Single amplified genome (SAG) technology separates individual cells before analyzing their DNA, thus giving us information about each cell’s genome instead of bulk metagenomes. SAG technology specifically allows whole-genome assemblies from small-sample volumes with low cell yields and low cell abundance compared to those of other cells within a given sample. This technology has proven to be especially useful for studying extremophiles, as their environment makes their cultivation and consequent genomic study particularly complicated (Karan et al., 2019). As the coherent successor of the SBM approach, SAG solved some bottlenecks by introducing a cell-sorting step, making the subsequent sequence analysis of a complex sample easier and more straightforward (Grötzinger et al., 2014). Although SBM and SAG technologies have been available for years, the expected boost in biotechnology has not been realized (Martin and Vandenbol, 2016). Despite the progress in next-generation sequencing technologies, relatively few new extremozymes have been discovered and functionally characterized using culture-independent methods. This shortfall is mainly, in addition to the high sequencing costs, because of the lack of reliable, functional annotation of the genomic data caused by the low amount (0.09%) of experimentally described genes (Grötzinger et al., 2014, 2017; Attrill et al., 2019).

Generally, annotation algorithms depend on existing functional annotations, thus giving reliable results for already well-studied branches of life.

Therefore, culture-based methods are still needed to study a novel enzyme’s function and structure, confirmed by several studies (Martin and Vandenbol, 2016; Speda et al., 2017).

Nevertheless, the lack of a specific database limits our choice for the right expression model to study the enzyme of interest. Consequently, the combination of these issues creates a vicious loop resulting in low detection and usage of extremozymes. This review aims to highlight the importance of extremozyme from prokaryotes research for industry by showing how recent developments in sequencing, computation and bioinformatics solve the problems associated with data mining for extremozymes and outline the annotation difficulties, which must be overcome in the future.

## Enzymes: A Sustainable Source for Green Chemistry

Enzymes are broadly used in biotechnology and a variety of industries (e.g., agriculture, food, textiles, chemicals, pharmaceuticals, and biofuels) as catalysts, therapeutic agents, analytic reagents, and diagnostic tools (Illanes et al., 2012; Robinson, 2015; Ramesh et al., 2020). Enzymes have broadened the horizon of potential applications by allowing us to perform chemo- and regioselective reactions, which is a big struggle for current chemical techniques (Rasor and Voss, 2001; Schäfer et al., 2007). In general, enzymatic reactions are safer, faster, less hazardous, and generate less waste, thus following the twelve rules of green chemistry (Anastas and Eghbali, 2010). Especially today, in a world with 7.5 billion people and an expected 9.8 billion 30 years from now (United Nations, Department of Economic and Social Affairs, Population Division, 2017), it is crucial to utilize the high potential of enzymes for biotechnological applications and green chemistry to reduce humanity’s overconsumption of resources (Anastas and Eghbali, 2010; Sheldon, 2016).

Although enzymes have vast potential in biotechnological applications, they have been used only in very few specific reactions. Most described enzymes can be used only for a limited number of industrial processes (Herbert, 1992; Elleuche et al., 2014). This limitation is caused by the narrow ranges of enzymatic stability, including a majority of essential parameters for chemical reactions, such as temperature, pressure, pH, and the use of organic solvents. For example, although water is considered the solvent of life, it is a poor solvent for synthetic reactions (Aitken and Brown, 1969). Organic solvents are used not only to increase the solubility of hydrophobic substrates but also to shift the thermodynamic equilibrium from hydrolysis to condensation and suppress water-dependent side reactions (Carrea and Riva, 2000).

A number of genetic (Gatti-Lafranconi et al., 2010) and chemical (Siddiqui et al., 2009) modifications together with immobilization strategies (Mukhopadhyay et al., 2015) have been developed to overcome these restrictions (Stepankova et al., 2013). These strategies aim to increase the enzyme’s stability or decrease the denaturing effect of the reaction conditions. To date, there is no ultimate mechanism to increase the stability of enzymes. A point mutation can drastically affect the enzyme’s efficiency and stability, although it is difficult to see any apparent trends or patterns to fully control a given enzyme (Vieille and Zeikus, 2001; Sarmiento et al., 2015). It is essential to identify mutagenesis strategies to alter enzymes from mesophilic organisms to achieve stability in extreme physicochemical conditions, which is a challenging task (Reed et al., 2013). Although various stabilizing immobilization strategies exist, several hurdles, such as a limited increase in stability, change in reaction rate, alteration of stabilities under other conditions, and potential influence on the specificity of the enzymes, have not allowed these approaches to be used in a broader range (Coker, 2016).

## Extremophiles

**FIGURE 1:**
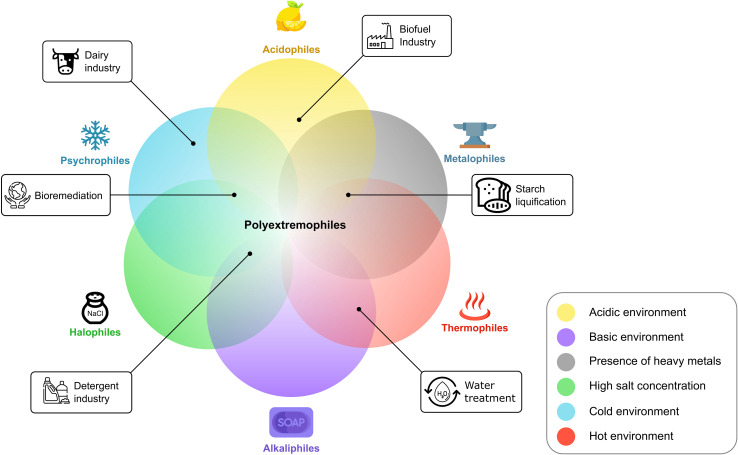
Selection of different extremophiles. The corresponding image displays the extreme condition(s) in which they thrive. Overlaps are examples of potential polyextremophiles and are not limited to the displayed configurations.

An alternative approach to achieve biocatalysis in extreme physicochemical conditions is to use enzymes derived from organisms that thrive in extreme conditions (Jorquera et al., 2019). These organisms are called extremophiles and live in harsh environments of elevated temperatures (thermophiles), salt concentration (halophiles), pressure (barophiles), osmotic compound content (osmophiles), heavy metal content (metalophiles), and radiation (radiophiles); acidic or basic pH (acid or alkaliphiles); extreme dryness (xerophiles); extreme cold (psychrophiles); or a combination of different extremes (polyextremophiles) (Figure 1; Kristjánsson and Hreggvidsson, 1995; Gerday and Glansdorff, 2009; Gabani and Singh, 2013; Coker, 2016; Horikoshi, 2016).

### Halophiles and Xerophiles

Halophiles are microorganisms that require elevated salt concentrations to grow. There are four main classifications of halophiles based on their optimal NaCl concentration for growth: slight halophiles (0.2 M), moderate halophiles (0.5–2.5 M), borderline extreme halophiles (2.5–4.0 M), and extreme halophiles (4.0–5.9 M). Halophilic organisms use several adaptation mechanisms to live in these conditions. These include modified electrostatic charge of their proteins, balancing osmotic pressure either by compatible solutes, such as betaine and ectoines, or with chloride and potassium uptake into the cells by transporters (primary or secondary) and the coordinated action of bacteriorhodopsin and ATP synthase (Karan et al., 2012a).

Halophilic organisms and their enzymes are used in several different fields, including production of fermented foods, manufacturing of solar salt from seawater, leather industries, environmental bioremediation and textile, pharmaceutical (Karan and Khare, 2010; Oren, 2010; Karan et al., 2012a; de Lourdes Moreno et al., 2013; Akal et al., 2019). The biochemical properties of the molecules, enzymes, and compatible solutes synthesized by halophiles also present potential implications in fine chemicals, medicines, and bioimplants (Irwin, 2010; Karan et al., 2012a, b; Chen and Jiang, 2018; Jin et al., 2019). One of the main selling points of these enzymes is their stability in such solvents as benzene, toluene or chloroform, which are frequently used in different industrial environments (Oren, 2010). Halophiles are a potential source of novel extremozymes like amylases, proteases, nucleases, cellulases, chitinases, xylanases, esterases, alcohol dehydrogenases and lipases (Karan et al., 2012a; Coker, 2016). For example, nuclease from *Micrococcus varians* has long been used commercially for production of a flavoring agent 5′-guanylic acid (5′-GMP) due to its efficiency in degrading RNA at 60°C and 12% (w/v) salt (Kamekura et al., 1982). Several other enzymes have been heterologously expressed and characterized (Table 1). Interestingly, some of the halophilic enzymes display polyextremophilicity i.e., stability toward more than one extreme condition e.g., high salt, elevated or low temperature, alkaline or acidic pH and non-aqueous medium having great potential application for industrial and biotechnological processes (Grötzinger et al., 2017; Akal et al., 2019; Karan et al., 2020).

**TABLE 1:** List of extremozymes discovered with metagenomics and single amplified genomes.

Enzyme	Source	Heterologous host	Screening approach
**Function-based metagenomics (FBM)**			
***β*-Glucosidase**	Hydrothermal spring	*E. coli*	Schröder et al., 2014
**Carboxylesterase**	Hot vent sediment	*E. coli*	Placido et al., 2015
**Esterase**	Glacier soil	*E. coli*	De Santi et al., 2015, 2016a,b
**Esterase**	Hot spring water, Sediment, and compost	*E. coli, Thermus Thermophilus*	Leis et al., 2015
**Esterase**	Hot spring water	*E. coli*	López-López et al., 2015
**Hormone-sensitive lipase**	Permafrost	*E. coli*	Petrovskaya et al., 2016
**Lipase**	Hot spring sediment	*E. coli*	Sahoo et al., 2017
**Lipase**	Hot spring water	*E. coli*	Yan et al., 2017
**Phospholipase**	Hydrothermal vent	*E. coli*	Fu et al., 2015
**Serine protease**	Hot spring sediment	*E. coli*	Singh et al., 2015
**Sequence-based metagenomics (SBM)**			
**α-Galactosidase**	Hot spring water	*E. coli*	Schröder et al., 2014
**Endoglucanase**	Hot spring sediment	*E. coli*	Zhao et al., 2017
**β-Galactosidase**	Hot spring water	*E. coli*	Liu et al., 2015
**Esterase**	Hot spring mud	*E. coli*	Zarafetaet al., 2016
**Mercuric reductase**	Brine pool	*E. coli*	Sayed et al., 2014
**Amine transferase**	Hot spring	*E. coli*	Ferrandi et al., 2017
**Epoxide hydrolase**	Hot spring	*E. coli*	Ferrandi et al., 2015
**Single amplified genomes (SAG)**			
**Alcohol dehydrogenases**	Brine pool	*Hfx. volcanii*	Grötzinger et al., 2017; Akal et al., 2019
**Carbonic anhydrase**	Brine pool	*Halobacterium* sp. NRC-1	Vogler et al., 2020
**Glucose dehydrogenase**	Brine pool	*Halobacterium* sp. NRC-1	Grötzinger et al., 2014; Karan et al., 2019
**2-hydroxy dehydrogenase**	Brine pool	*Halobacterium* sp. NRC-1	Grötzinger et al., 2014; Karan et al., 2019
**Protease**	Brine pool	*Halobacterium* sp. NRC-1, *E. coli*	Grötzinger et al., 2014; Karan et al., 2019

Along with the enzymes, halophiles possess a number of bioactive molecules that found its application in different areas. The biocompatible solute ectoine has potential use in respiratory medicine (Galinski et al., 1985) and can reduce cell stress effects in nanoparticle-induced lung inflammation by inhibiting the signals (Sydlik et al., 2009). Some halophiles, such as *Halobacterium* and *Haloferax*, were found to accumulate polyhydroxyalkanoates (PHAs), which are a family of biopolyesters with diverse uses in the medical, environmental, and industrial fields (Kirk and Ginzburg, 1972; Quillaguaman et al., 2010; Chen and Patel, 2012). Halophiles, such as *Natronococcus occultus* and *Naloterrigena hispanica*, have been found to generate antimicrobial peptides and diketopiperazines (Charlesworth and Burns, 2015; Coker, 2016). Some haloarchaea produce internal protein gas vesicle nanoparticles (GVNPs), which have been tested as a vaccine scaffold platform and ultrasonic contrast agents (Stuart et al., 2001; Shapiro et al., 2014; DasSarma and DasSarma, 2015). Notably, halophilic nature makes cell lysis, hence farming of the enzymes and other molecules, easier. Since halophiles require high salt to maintain their cell integrity, lowering the salt concentration in the buffer system disrupts the cell wall of most halophiles.

Xerophiles are microorganisms that can survive and grow in arid conditions with water activity *a*_*w*_ < 0.75 by forming spores that help them mitigate environmental stress. Adaptive mechanisms are connected to water loss prevention and increased water retention through the accumulation of compatible solutes, production of extracellular polymeric substances (EPSs), adaptations on the cell membrane to retain intracellular water, and synthesis of DNA repair proteins (Lebre et al., 2017). These unique adaptations allow xerophiles to be used in microbial electrochemical systems (Dopson et al., 2015) or in next-generation industrial biotechnology, where they can be used for treating long-chain fatty acids, cellulose, chitin, rubbers, or other compounds (Chen and Jiang, 2018).

### Thermophiles and Psychrophiles

Thermophiles are heat-loving microorganisms with an optimum growth temperature of 45°C or above, while hyperthermophiles grow at temperatures above 80°C (Sarmiento et al., 2015). Thermophiles and hyperthermophiles exist in various natural ecosystems, such as geothermal waters, hot springs, volcanoes, deep-sea hydrothermal vents, and other ecosystems with high-temperature parameters. Thermophiles have thermostable proteins and cell membranes that do not become denatured at high temperatures, and some may also resist proteolysis (Sarmiento et al., 2015). Notably, polymerases from thermophiles have laid the foundation for the discovery of polymerase chain reaction (PCR), a technique that has become crucial in medicine and research. Today we may find various enzymes from thermophiles (e.g., from *Thermus aquaticus* and *Pyrococcus furiosus*) finding their use in PCR due to their stability and reasonable cost (Brock, 1997; Bruins et al., 2001; Irwin and Baird, 2004). Along with polymerases, a lot of different thermophilic enzymes, such as lipases, laccases, and xylanases, are also on the market, thus making the industrial process much more environmentally friendly (Damiano et al., 2003; Atalah et al., 2019).

Psychrophiles are cold-loving microorganisms that can grow at temperatures between −20 and 20°C. Psychrophiles possess diverse adaptive molecular mechanisms to survive and thrive at such low temperatures. Psychrophilic bacteria have increased (i) unsaturated fatty acids, cyclopropane-containing fatty acids, and short-chain fatty acids in their membranes, which prevent the loss of membrane fluidity; (ii) cold-shock proteins (CSPs) and chaperones to protect the synthesis of RNA and proteins; (iii) antifreeze proteins (AFPs) that bind to ice crystals and create a state of thermal hysteresis; and (iv) mannitol and other compatible solutes that act as cryoprotectants to prevent cell damage by ultraviolet (UV) radiation and ice formation (Sarmiento et al., 2015; De Santi et al., 2016a).

Psychrophiles have a promising future in pharmaceuticals and medicine since their cell membranes hold surfactants capable of sustaining stability at low temperatures (Cavicchioli et al., 2011). Psychrophiles, such as *Pandalus borealis*, *Euphausia superba*, *Moraxella* species, and *Flavobacterium* species, have been found to produce anticancer and antitumor agents (Margesin and Feller, 2010). Psychrophilic enzymes display high catalytic activity, stability at low temperatures, and pronounced heat lability and may offer useful industrial and biotechnological applications in various domains, such as pharmaceutical science, molecular biology, textiles, paper, food, feed technologies, detergents, and cosmetics (Margesin and Feller, 2010; Cavicchioli et al., 2011; Sarmiento et al., 2015; Dhaulaniya et al., 2019; Al-Ghanayem and Joseph, 2020).

### Acidophiles and Alkaliphiles

Acidophiles are microorganisms that grow at an optimum pH < 3 (Baker-Austin and Dopson, 2007). Acid-tolerant microbes have optimum growth at pH > 5 but are still active in lower pH environments. Acidophiles maintain their cytoplasmic pH close to neutrality to protect acid-labile cellular constituents by active pumping of protons (proton flux system); by decreased permeability of the cell membrane, which helps to suppress the entry of protons into the cytoplasm; and by improved protein and DNA repair systems compared to those of neutrophiles (Baker-Austin and Dopson, 2007). Acidophilic enzymes can block the activity of matrix metallopeptidases (MMPs), which are essential for tumor metastasis (Irwin, 2010). MMP inhibitors from an acidophilic *Penicillium* species isolated from Berkeley Pit Lake promise a therapeutic approach for cancer (Stierle et al., 2006). Proteolytic enzymes from acidophiles have also been reported as nonallergenic preservatives in medicines (Sharma et al., 2012). Amylolytic enzymes, such as trehalase isolated from acidophilic *Sulfolobus solfataricus*, are used in medicine as preservatives and stabilizers (Schiraldi et al., 2002).

Alkaliphiles are microorganisms that grow in alkaline environments with a pH > 9, usually showing optimal growth at pH ∼10. Alkaliphiles may coexist with neutrophiles under mild basic pH conditions and live in specific extreme environments. Alkaliphilic bacteria possess molecular mechanisms that compromise the activation of both symporter and antiporter systems. Electrogenic antiporters produce an electrochemical gradient of Na^+^ and H^+^, and the symporter system enables the uptake of Na^+^ and other solutes into the cells (Chinnathambi, 2015). Alkaliphilic enzymes have found use in several applications, such as tannery water treatment (Li et al., 2017), food, cosmetics, and pharmaceutical production (Horikoshi, 2016).

### Radiophiles

Radiophiles or radiation-resistant extremophiles thrive in high oxidative stress and radiation environments, including UV radiation, gamma radiation and X-ray radiation, and have potential applications in therapeutics pharmacology and biotechnology. Direct intense or prolonged exposure to different forms of radiation, such as UV radiation, can lead to mutagenic and cytotoxic DNA lesions, resulting in various types of human cancers (Gabani and Singh, 2013). Primary and secondary metabolic products from radiophiles can protect the organism’s DNA and can be used to manufacture anticancer drugs, antioxidants, and sunscreens (Raddadi et al., 2015). Extremolytes, mycosporin-like amino acids (MAAs) from the red alga *Porphyra rosengurttii*, are commercially utilized to enhance the UV-protective properties of sunscreens (de la Coba et al., 2009) and are therapeutic candidates as preventive agents in UV radiation-induced cancers, such as melanoma. Bacterioruberin isolated from *Halobacterium* and *Rubrobacter* and deinoxanthin isolated from *Deinococcus radiodurans* are other therapeutic candidates for cancer diseases (Singh and Gabani, 2011; Choi et al., 2014).

### Polyextremophiles

Polyextremophiles i.e., microorganisms growing preferentially under multiple extremes, have developed features that allow them to thrive in harsh environments such as Deep Lake Antarctica, where the temperature reaches as low as −20°C and stays liquid only due to extreme salt concentrations (DasSarma et al., 2013; Karan et al., 2013, 2020). Deep-sea anoxic brine pools at the bottom of the Red Sea are another type of polyextremophilic environment (Antunes et al., 2011). These brine pools are extreme in different physicochemical parameters and vary drastically, with temperatures ranging from 22.6 to 68.2°C and NaCl concentrations varying from 2.6 to 5.6 M (Antunes et al., 2011). Additionally, they show a characteristic sharp brine-seawater interface, with steep gradients of dissolved oxygen, density, pH, salinity, and temperature (Emery et al., 1969; Ross, 1972; Anschutz and Blanc, 1995). Because of this variation, brine pools offer a multitude of habitats for different kinds of extremophiles.

Polyextremophiles have a diverse range of uses and applications. Halothermophiles and halopsychrophiles are promising sources of useful enzymes (Elleuche et al., 2014). Enzymes derived from these polyextremophilic microbes possess particularly attractive properties for biotechnology, namely, their function at high salt concentrations and high or low temperatures (Sarmiento et al., 2015). Elevated temperatures are used to shift equilibria, distill products, increase reaction’s speed, liquefy compounds, and eliminate microbial contamination (Grötzinger et al., 2017). Low temperatures can save energy (e.g., in washing processes, bioremediation, or food processing), avoid labile or volatile compound production (e.g., in biotransformations or food processing) or prevent bacterial growth. Therefore, cold-active enzymes have great potential for various biotechnological processes. Increasing the salt concentration in solution decreases the water activity, thus mimicking aqueous-organic solvent mixtures. Therefore, halophilic enzymes generally retain high activity and stability in high salt and organic or nonaqueous media (Karan and Khare, 2010, 2011; Karan et al., 2011, 2012a). In turn, organic solvents increase the solubility of hydrophobic substrates and alter the hydrolytic and kinetic equilibria (Sellek and Chaudhuri, 1999). Traditional mesophilic enzymes lose their native structure and thus catalytic activity in organic solvents, limiting their use. However, the high stability of halophilic enzymes toward salt is associated with tolerance to low water activity, such as in mixtures of aqueous and organic or nonaqueous media (Sellek and Chaudhuri, 1999), which emphasizes their high potential for biocatalysis. Cold-active or heat-stable enzymes operating in high-salt or organic solvents are of interest for the sustainable production of value-added chemicals. The world is currently looking for microorganisms with new enzymes, such as hydrolases, amylases, cellulases, peptidases, and lipases (Dumorne et al., 2017). Some examples include alkalithermophilic serine proteases from *Alkalibacillus* sp. NM-Da2, which can potentially be applied in different biotechnological and pharmaceutical industries (Abdel-Hamed et al., 2016), and alkalipsychrophilic esterase from the marine bacterium *Rhodococcus* sp., which can be used in the food industry since the process catalyzed by an esterase can be stopped by increasing the temperature, thus saving food properties (De Santi et al., 2014). The polyextremophilic characteristics of halophilic amylases potentially make them efficient catalysts under alkaline pH and high salinity in processes such as starch hydrolysis and applications such as detergent production, the food industry, and bioremediation (Ali et al., 2014; Kumar et al., 2016; Rekadwad and Khobragade, 2017).

## Mining Enzymes From Extreme Environments

Enzymes derived from extremophiles, so-called extremozymes, can catalyze chemical reactions in harsh conditions, such as those found in industrial processes (Sarmiento et al., 2015). The current global market for industrial enzymes was 9.9 billion USD in 2019 and is expected to grow to 14.9 billion USD by 2027 (Grand View Research, 2020)^1^, in which novel extremozymes could play a significant role and further expand this market.

Extremophiles thrive in extreme habitats, including salt lakes, deep-sea vents, acidic sulfurous lakes, alkaline lakes, hot springs, Arctic and Antarctic waters, and alpine lakes (Kristjánsson and Hreggvidsson, 1995; Gerday and Glansdorff, 2009). The challenges in mining the enzymatic potential of extremophilic locations are the hurdle of mimicking these harsh conditions in the laboratory (Sarmiento et al., 2015), the remote areas, and the unusually low cell density, causing a minimal amount of biomass yields (Ferrer et al., 2007). As a result, despite the high scientific potential and industrial value of extremozymes, very little is known about their structure and function. Therefore, most enzymes currently used in industry originate from either fungi or mesophilic bacteria (Elleuche et al., 2014).

### Microbial Dark Matter—The Big Unknown

According to recent estimations, Earth is home to an upward range of 10^12^ (1 trillion) microbial species, with microorganisms being the most abundant, widespread, and taxonomically, metabolically, and functionally diverse organisms (Locey and Lennon, 2016). During the past decade, high-throughput sequencing in combination with advanced bioinformatics algorithms has allowed for enhanced insight into microbial taxa and expanded the estimation of the global microbial load by orders of magnitude via projects such as the “Earth Microbiome project” (EMP), which analyzed more than 200,000 environmental samples (Gilbert et al., 2014). Out of the 10^12^ microbial species, only approximately 10^5^ have been sequenced (0.00001%), 10^4^ have been cultured, and even the EMP, which also uses rRNA sequences for identification, has cataloged fewer than 10^7^ species (0.001%), of which 29% were detected only twice (Locey and Lennon, 2016). It is estimated that approximately 99.999% of microbial taxa remain undiscovered (Locey and Lennon, 2016). Focusing on the ∼10^4^ cultivated species and looking at the highest order, among the 60 significant lines of descent (phyla or divisions) that are known within the archaeal and bacterial domain (Hugenholtz and Kyrpides, 2009), 50% are uncultured and make up the “microbial dark matter” (Marcy et al., 2007). Furthermore, 88% of all isolated microbes are members of only four bacterial phyla, *Proteobacteria, Firmicutes, Actinobacteria*, and *Bacteroidetes* (Rinke et al., 2013).

### The Challenge of Cultivation

Traditional cultural methods employ the ability of microorganisms to grow in a specific laboratory environment. Historically, these methods have been used in microbiology, including selective or differential media, microscopy, Gram-staining, and biochemical tests. These methods are sensitive, reliable, inexpensive, and provide qualitative and quantitative results on the bacterial populations (Nowrotek et al., 2019). However, only a small fraction of all microbes can be grown in a laboratory setting. Thus, although cultural methods are a well-established, simple, and inexpensive way of isolating, detecting, and quantifying microorganisms, the methods lack speed and are labor-intensive with the risk of contamination. Based on this legacy, microbial culturomics has emerged as a tool that complements metagenomics data and gives another way to determine composition of microbial populations. Several studies have shown that culture-dependent and culture-independent methods often deliver different results (Steven et al., 2007; Carraro et al., 2011; Stefani et al., 2015; Nowrotek et al., 2019; Rego et al., 2019). For example, using 212 different culture conditions, such as temperature, various oxygen levels, and selective media, many researchers have isolated 340 different bacterial species (Lagier et al., 2012). Interestingly, the metagenomics approach could identify only 51 species out of 340 cultured species in this study. Therefore, it is crucial to combine both culture-dependent and culture-independent ways to study a given microbiome (Steven et al., 2007; Lagier et al., 2016; Nowrotek et al., 2019; Sarhan et al., 2019).

The enormous lack of isolates is based on the fact that very few microbes are cultivable, e.g., only as few as 0.001–0.1% of the microbes found in seawater can be cultivated under laboratory conditions (Amann et al., 1995). Therefore, archaea enzymes are typically studied after expression in a heterologous host, mostly *Escherichia coli* (*E. coli*). However, this approach is impossible for some archaeal proteins due to low expression rates, inaccurate protein folding, or the lack of functionally necessary posttranslational protein modifications in eubacterial hosts. Besides, it is challenging to identify archaeal enzymes directly from genome samples in bacterial host-based functional screening assays, as archaeal promoter structures differ from bacterial ones (Zweerink et al., 2017). A direct *in vivo* investigation of archaeal enzymes in an archaeal strain or an archaeal host with the right expression machinery of the respective proteins represents a valuable alternative. For this reason, some archaeal expression models have been used, such as *Halobacterium salinarum*/NRC-1 or *Haloferax volcanii*. Using these expression systems, researchers have studied archaeal TATA-binding proteins, discovered 16S gene transfer between bacteria and archaea (Baliga et al., 2000; Fuchsman et al., 2017), and several different archaeal enzymes have been studied using these archaeal expression systems (Table 1). However, this approach requires technically sensitive and straightforward technologies to detect active enzymes and a methodology to express proteins in an archaeal host heterologously. Ultimately, as extremophiles preferentially live in harsh conditions, it is difficult to satisfy all of these microbes’ extreme requirements. However, with the progress in computational methods and improving bioinformatics algorithms, it is now possible to study the mysterious “dark matter” of the microbial world using culture-independent approaches, including bulk metagenomics and SAGs.

### Function-Based Metagenomics (FBM), Sequence-Based Metagenomics (SBM), and Single Amplified Genomes (SAGs) as a Gateway to Novel Species

Culture-independent methods developed and utilized over the last few years, such as metagenomics, have recently gained momentum. Metagenomics analyses rely on the direct isolation of genomic DNA from the environment. These analyses can be either sequence-based (i.e., putative enzymes are discovered based on their conserved sequences) or function-based (i.e., functional enzymes are found based on the expressed features, such as a specific enzyme activity).

#### Function-Based Metagenomics (FBM)

The function-based metagenomics (FBM) approach is based on cloning random environmental DNA into expression hosts, such as *E. coli*, forming a library. This library is then screened for function by different assay methods (Rashid and Stingl, 2015). The main challenge of this method is the need for hosts to create functional expression libraries and sufficient screening methods (Rashid and Stingl, 2015). The alternative is the sequence-based approach, which historically was based on sequence homology and used a colony hybridization technique to screen metagenomic clones using an oligonucleotide primer or probes for the target gene. In a subsequent step, the desired gene was amplified by PCR and cloned into suitable expression vectors. This technique led to discovering novel sequences similar to existing known sequences and may help find enzymes more efficiently (Raghunathan et al., 2005; Marcy et al., 2007).

However, metagenomics approaches give little information about expression and regulation of genes in the environment. Recently, metatranscriptomics approaches have been used to reveal gene expression profiles and ecophysiology of natural microbial communities (Hua et al., 2015; Tripathy et al., 2016). Combined genomic and transcriptomic analyses can discover and characterize the relative transcriptional levels of multiple genes and disclose the functional diversity in microbial communities. Such approach already has been utilized for studying extremophiles in acidophilic conditions and provided important data on *Ferrovum* population and its metabolic potentials and gene expression profile (Tripathy et al., 2016). In the end, metatrascriptomics approach allowed researchers to peak into the ecological role of these unexplored, but potentially important microorganisms.

#### Sequence-Based Metagenomics (SBM)

**FIGURE 2:**
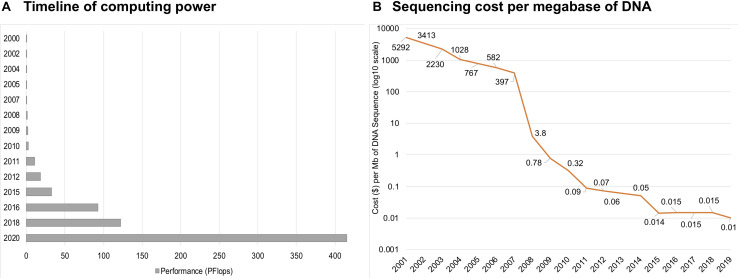
The timeline of computing power shows a dramatic increase over the last two decades **(A)**. Sequencing cost per megabase (Mb; one million bases) of DNA in USD **(B)**.

SBM became more attractive approach with the advent of high-throughput sequencing, when the main biases and bottlenecks of environmental sampling along with the requirement of DNA cloning before sequencing were removed (Bohmann et al., 2014). Today, the DNA from metagenomic samples can be sequenced without cloning. Furthermore, sometimes it is possible to extract desired gene sequences directly from metagenomics data and synthesize the genes *de novo* after bioinformatic annotation and codon optimization if required. These developments shifted the bottleneck of culture-independent sequencing, mainly to bioinformatics-based sequence alignment, data management, and, in particular, annotation algorithms. Annotation algorithms initially relied on sequence homology-based methods and, therefore, depended on existing functional annotations, giving excellent results only for already well-studied life branches. Therefore, it is advisable to use additional methods to account for this point. Different annotation approaches arose over time, still based on existing functional annotations but offering more degrees of freedom regarding the DNA sequences. Big data analysis requires tremendous computing powers, and for a long time, computers were not ready to handle that kind of load (Fan et al., 2014). Since introduction of the first supercomputer in 1964, the performance has dramatically increased, and now the fastest supercomputer is more than 100 times more powerful than those from a decade ago (Figure 2A). Along with the development of powerful computers and advanced computing algorithms, the cost of sequencing has been continuously decreasing, and in the last two decades, it has dropped by a factor of over one million (Wetterstrand, 2013; Figure 2B). Increased availability opened up multiple possibilities to study genomes, and several technologies appeared, including first-, second-, and third-generation sequencing. Due to the rapid development of next-generation sequencing, shotgun and high-throughput versions of SBM have become more popular. The SBM method allows for the sequencing of environmental DNA (Thomas et al., 2012) by randomly shearing all metagenomic DNA from one sample, a method depending on the cloning step into vectors for amplification, and sequencing the vector DNA, followed by assembly via algorithms (Rashid and Stingl, 2015).

Despite many benefits, SBM also suffers from limitations and challenges. Not only does the short read length of the DNA sequencing and the size and complexity of the sequence data pose analytic and informatics challenges (Rashid and Stingl, 2015), but also the ability to assemble independent genomes is drastically reduced at both very low abundances and increasing genomic heterogeneity (Albertsen et al., 2013). These challenges result in a low probability of identifying rare populations, occurring in less than 1% of the total metagenomic DNA (Kunin et al., 2008). This effect comes mainly from cross-strain assemblies (Rinke et al., 2014) that originate from different organisms but can also come from unwanted host DNA or environmental contamination (Schmieder and Edwards, 2011) that cannot be avoided. Hence, despite high expectations, metagenomics data have not yet led to the expected boost in biotechnology (Chistoserdova, 2010).

#### Single Amplified Genomes (SAGs)

**FIGURE 3:**
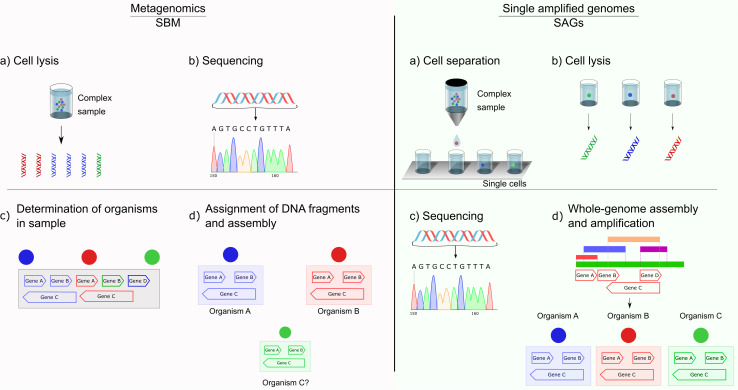
Schematic comparison of sequence-based metagenomics (SBM) and single amplified genomes (SAGs). In contrast to the multitude of sequences obtained in metagenomics, SAG allows the correct assignment of one genome to one sample. Different amounts of diverse species result in an uneven distribution of DNA fragments for metagenomics, drastically increasing complexity in the assignment of DNA fragments to potential genomes and their assembly.

SAGs gain the momentum due to the breakthrough of single-cell genome sequencing that has alleviated many bottlenecks in metagenomics by physically separating the genomic material of uncultured cells (Raghunathan et al., 2005; Kvist et al., 2007; Marcy et al., 2007). The Figure 3 shows a schematic comparison of the critical steps in SBM and SAGs screening using SAGs. In recent years, single-cell genome sequencing has become a highly accessible tool (Chi, 2014). In part, this utility is due to the improvements that have taken place in the protocols for DNA or RNA isolation, leading to more simplified procedures. Additionally, several industrial kits have become available, leading to the amplification of genetic material from single cells (Chi, 2014). To prepare samples for further operations, they need to be separated from the bulk cell mass. There are several different methods for sample preparation of single cells for single-cell genomics (SCG) (Blainey, 2013). These methods include serial dilution (Zhang et al., 2006), micromanipulation (Woyke et al., 2010), optofluidics (optical tweezing in conjunction with microfluidics) (Landry et al., 2013), laser-capture microdissection of tissue samples (Frumkin et al., 2008) and fluorescence-activated cell sorting (FACS) (Swan et al., 2011; Dupont et al., 2012; McLean et al., 2013; Wilson et al., 2014), ultimately resulting in individually sorted samples enriched with a particular microbe into 96-well plates, with a minimal amount of either or both host and environmental contamination. FACS has become the most popular method due to its high performance and the ability to separate individual environmental cells based on various cellular properties (e.g., size, fluorescence, and granularity). Moreover, FACS can also be used to study populations (Thompson et al., 2013). The enriched pool of symbiont cells can undergo whole-genome amplification (WGA) followed by sequencing, yielding a population genome assembly or a homogeneous draft assembly in a clonal population. However, FACS has some technical problems for the sorting of microbial cells. Because it is impossible to confirm cell identity visually, sometimes non-cellular fluorescent particles present in environmental samples can be sorted along with targeted microbial cells (Davey and Kell, 1996; Müller and Nebe-Von-Caron, 2010). For the same reason, FACS also retains a low efficiency in recovering rare cells (Yamamura et al., 2005; Yoshimoto et al., 2013; Nakamura et al., 2016).

After sorting, cells need to be lysed (e.g., with an alkaline solution, detergent, or heat). Furthermore, to obtain DNA contigs, the sample undergoes multiple displacement DNA amplification to sequence genomic DNA from a single microorganism. Subsequently, microorganisms, or unamplified single DNA fragments can be screened in each well using PCR with specific gene primers. WGA can also be performed using bacteriophage Φ29 polymerase to identify specific genes in a genome. Finally, it is vital to monitor DNA contamination to ensure that all reagents and equipment used are decontaminated (Giddings and Newman, 2015).

### The Challenge of Gene Function Annotation

Although the accessibility of uncultured species’ genomes has increased, computational challenges hinder discovering novel extremozymes using culture-independent methods. These hindrances are not unique to extremophiles and affects all organisms, albeit every group has its own unique hurdles contributing to the major cause. The major challenge in mining uncultured organisms’ genomic data is the reliable large-scale annotation of DNA sequences from non-mesophilic organisms with low sequence homology to experimentally described organisms. Annotation by homology faces a significant dilemma: annotation reliability is reciprocally coupled to protein diversity. Therefore, the difference between gene sequences negatively correlates with the overall homology to any related gene. Moreover, studying extremozymes amplifies not only in culture-based approaches but also culture-independent methods. First reason is linked to the fact that extremophiles tend to alter the overall amino acid composition of enzymes to adapt to their environment, which can be observed in halophiles and thermophiles (Madern et al., 2000; Sterner and Liebl, 2001; Berezovsky and Shakhnovich, 2005; Radestock and Gohlke, 2011; Siglioccolo et al., 2011; Reed et al., 2013; Karan et al., 2020). The second reason is mainly ought to the very limited amount of characterized extremozymes. For example, alcohol dehydrogenases and γ-carbonic anhydrase (CA_D) discovered from uncharacterized archaea collected from brine pool at the bottom of the Red Sea showed sequence homology of about 30–37% to the nearest mesophilic homologs (Grötzinger et al., 2017; Akal et al., 2019; Vogler et al., 2020).

**FIGURE 4:**
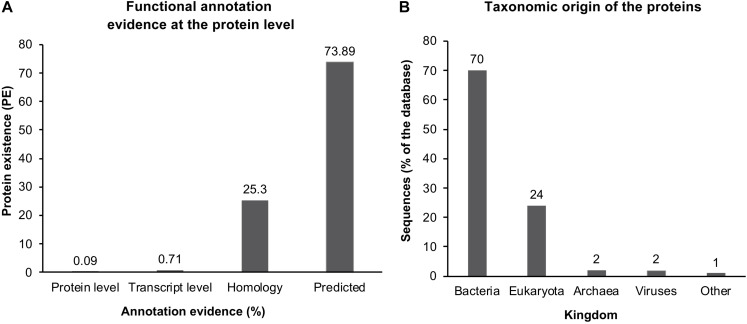
Protein sequence entries in the UniProt database (2020_03 release, https://www.ebi.ac.uk/uniprot/TrEMBLstats). Number of proteins annotated using the listed evidence of their functional annotation prediction from 185 million sequence entries. Only 0.09% of all entries, corresponding to ∼169 thousand entries, show any functional annotation evidence at the protein level **(A)**. Taxonomic origin of the proteins in percent, separated into Kingdoms **(B)** (data from https://www.ebi.ac.uk/uniprot/TrEMBLstats).

Error propagation further complicates the situation, especially in enzymes with few or highly diverged homologs. The function of the encoded protein can be proven experimentally only for a small and continuously decreasing fraction of gene sequences available from databases, which is currently 0.09% of the UniProt database (Figure 4A). Furthermore, more than 94% of all sequences originate from eukaryotes or bacteria (Figure 4B). Therefore, the challenge of a reliable annotation is even more pronounced for genes that originate from other organisms, such as archaea, including most extremophiles (Rampelotto, 2013). Hence, the more distinct a protein is, smaller the probability of being related to an experimentally described enzyme. At the same time, the more distinct an enzyme is, the higher is its impact on future annotations. For example, a gene from the newly discovered mesophilic microorganism can be compared to many homologs. The function of the majority of these homologs will be used as annotation of the new gene. In contrast, a gene from a newly discovered extremophilic microorganism might have far fewer homologs and therefore, the annotation of these homologs plays a significant role for future studies. Recent developments in annotation algorithms allow a higher degree of freedom between DNA sequences that will be annotated compared to the closest homologs. These methods include algorithms that focus on specific and short conserved sequences only, such as essential amino acids of the active center along with conserved regions such as catalytic sites and cofactor binding sites (Grötzinger et al., 2014). More elegant but substantially more complicated and computational resource-demanding are structure-based approaches. Here, the expression product of potential genes is modeled, and its function is assessed by its structure, not by the sequence. The biennial Critical Assessment of protein Structure Prediction (CASP) is a competition where their efficiency compares novel modeling algorithms to predict unusual and novel tertiary structures of proteins whose crystal and/or NMR structure was measured but not yet published. The overall *in silico* modeling success was limited until the rise of AI-based protein folding prediction. Last year, in 2020, a new version of a deep machine learning-based algorithm called AlphaFold 2 was released (Senior et al., 2020). This algorithm has substantially increased computational protein folding prediction potential and showed impressive results in the CASP assessment. AlphaFold 2 reached 92.4 out of 100 Global Distance Test (GDT), whereas the average crystal structure only gets 90. Thus, at least for the test samples, it was as good as a crystal structure. Such an impressive result gives hope for the future computational study of unknown proteins. In theory, it could mean that any given protein’s structure and function could be predicted solely based on its amino acid sequence in the future. Still, today only a limited amount of algorithms have experimentally been shown to be efficient in the annotation of far distant related genes (Grötzinger et al., 2014; Akal et al., 2019; Vogler et al., 2020). Reliable annotation of the entire genome of an organism that is very distantly related to described organisms is not yet available.

SAGs reduce data complexity significantly compared to that of the metagenomics approach, but the analysis and management of next-generation whole-genome sequencing (NGS) data include an impressive number of various software applications. These applications are used for sequence read assembly, mapping to the reference genome, variant/SNP calling and annotation, transcript assembly and quantification, and small RNA identification (Horner et al., 2010; Garber et al., 2011; Pabinger et al., 2013). Compared to classical genomic sequencing, experimental characterization of SAG gene products requires gene synthesis, expression, purification, and functional characterization and, therefore, is several orders of magnitude more time-consuming and cost-intensive. Hence, false-positive results from flawed annotation are much more problematic than false-negative results (due to incomplete annotation) when genomic data are searched for the desired function, which is particularly true for genes from extremophilic organisms that require slow-growing expression systems (Grötzinger et al., 2014).

Therefore, the initial challenge of cultivating organisms can be addressed to a considerable degree by using novel culture-independent methods. However, these advance shifts the problem toward bioinformatics-based handling and interpretation of the data, where developments are rapidly progressing but still need time and data, to allow reliable, functional annotation of whole genomes. In the Table 1, a comprehensive list of extremozymes discovered with metagenomics (Berini et al., 2017) and SAGs are shown.

## Conclusion/Outlook

In the past decades, DNA sequence analysis has made tremendous progress with advances in sequencing technologies and the rapid development of data analysis algorithms. Through these developments, metagenomic studies of complex microbial communities have become more straightforward and more applicable. Given that most microbes cannot be cultivated in a laboratory, obtaining genome information directly from the environment is a massive step forward for genome profiling of unknown microbial communities. At the time, when read lengths are long enough to allow a confident assembly of genomes, SBM will become the method of choice for bioprospecting non-cultivable microorganisms. For now, the implementation of single-cell genomics has facilitated recovering proteins from uncultured microorganism, helped us understand the species diversity, and how enzymes adapt to harsh environments. Recent advances in developing new sequencing technologies with longer reads, higher throughput, and better cell sorting methods have certainly enhanced SCG as a tool for studying uncultivable microbes. Consequently, this relatively cost-efficient technology is essential to broaden the knowledge of extremophiles and extremozymes, which are very distantly related to the majority of described organisms. We believe that single-cell technologies will not only shed further light on microbial “dark matter” but also facilitate the development of *in silico*-designed and fine-tailored biocatalysts for specific reactions, thus bringing us closer to a sustainable future.

## Author Contributions

All authors contributed to and commented on this manuscript.

## Conflict of Interest

The authors declare that the research was conducted in the absence of any commercial or financial relationships that could be construed as a potential conflict of interest.

## Footnotes

1 https://www.grandviewresearch.com/industry-analysis/enzymes-industry, last access date: 2/1/2021 4:43 p.m.
